# Comparative Evaluation of Sodium Bicarbonate and Hyaluronidase as Adjuncts to Lignocaine in Peribulbar Block During Cataract Surgery

**DOI:** 10.7759/cureus.90630

**Published:** 2025-08-20

**Authors:** Sanchita Gupta, Abha Gahlot, Divya Singh, Charu Malik, Rishabh S Kushwaha, Jawahar Goyal

**Affiliations:** 1 Ophthalmology, School of Medical Sciences and Research, Sharda University, Greater Noida, IND

**Keywords:** akinesia, cataract surgery, hyaluronidase, lignocaine, peribulbar anesthesia, sodium bicarbonate

## Abstract

Background

Peribulbar anaesthesia is widely used in cataract surgery to provide reliable analgesia, akinesia, and a low rate of complications. Hyaluronidase is conventionally added to local anaesthetic solutions to enhance diffusion and improve block quality. More recently, sodium bicarbonate has been suggested as an alternative adjunct, as its alkalinising effect increases the proportion of non-ionised lignocaine, potentially accelerating onset and improving efficacy. This study aimed to compare the effectiveness of sodium bicarbonate and hyaluronidase as adjuncts to lignocaine in peribulbar block during cataract surgery, with particular focus on pain perception, onset of anaesthesia, and onset of akinesia.

Materials and methods

A randomised interventional study was conducted on 80 patients undergoing cataract surgery, divided equally into two groups of 40. Group A received a mixture of lignocaine, bupivacaine, and hyaluronidase, while Group B received lignocaine, bupivacaine, and sodium bicarbonate. Pain intensity was assessed using the Visual Analogue Scale (VAS), and the onset of anaesthesia and akinesia were recorded at one-minute intervals for up to 10 minutes. Data were analysed using IBM SPSS Statistics for Windows, Version 22.0 (IBM Corp., Armonk, NY, USA), with statistical significance set at p < 0.05.

Results

Patients in Group B reported significantly lower pain scores (p < 0.05) and a faster onset of anaesthesia compared to Group A. In contrast, the onset of akinesia was significantly slower in Group B. Complication rates were comparable between the two groups, with no statistically significant differences observed.

Conclusion

The findings suggest that sodium bicarbonate is a useful adjunct to lignocaine in peribulbar anaesthesia, offering faster onset and reduced pain perception, though its effect on akinesia requires further evaluation.

## Introduction

The prevalence of blindness due to cataract (pinhole visual acuity <3/60 in the better eye with lens opacity in both eyes) in India is 0.84%, while the prevalence of bilateral cataract-related visual impairment (pinhole visual acuity <6/18 in the better eye) is 5.09%. Cataract remains the leading cause of blindness in India, accounting for 66.21% of cases [1]. Cataract surgeries are now commonly performed under local or topical anaesthesia, which has reduced the use of general anaesthesia and its associated side effects. The primary goal of anaesthesia is to ensure patient comfort and provide optimal surgical conditions. It should achieve akinesia, analgesia, anaesthesia, minimal bleeding, and avoidance of complications such as raised intraocular pressure, oculocardiac reflex, globe penetration, intraocular trauma, or inadvertent brainstem anaesthesia.

Peribulbar anaesthesia was first described in 1985 by Davis and Mandel, where the anaesthetic solution is injected into the peripheral orbital space rather than the central intraconal space, as in a retrobulbar block. This approach reduces the risk of injuring orbital structures [2]. Historically, cocaine was the first agent used for ocular anaesthesia, introduced by Markel for enucleation [3].

The anaesthetic solution routinely used contains hyaluronidase along with 2% lignocaine and 0.5% bupivacaine. Lignocaine provides a rapid onset but shorter duration of action, whereas bupivacaine has a slower onset with prolonged effect. Hyaluronidase improves the diffusion of the solution. It is an enzyme that breaks down hyaluronic acid, the most abundant glycosaminoglycan in connective tissue, into smaller fragments by hydrolysing the hexosaminidic linkage of the disaccharides [4]. This enhances drug diffusion and tissue permeability.

In contrast, the addition of sodium bicarbonate increases the pH of the anaesthetic mixture, a process referred to as “alkalinisation.” Galindo reported that this modification reduces the onset time and improves the spread of anaesthesia by raising the pH [5]. According to the Henderson-Hasselbalch equation, the ratio of ionised to non-ionised fractions depends on both the pKa of the drug (lignocaine = 7.9) and the pH of the solution. Thus, the addition of sodium bicarbonate to lignocaine increases the non-ionised fraction, facilitating more effective diffusion across nerve membranes [6].

The alkaline form of the drug is active, and alkalinisation with sodium bicarbonate increases the non-cationic fraction, resulting in faster drug infiltration. It also reduces pain on infiltration [7], which can be attributed to the faster onset of action rather than a mere change in pH [8].

Only a few studies have examined the effect of lignocaine alkalinisation on pain during peribulbar block in cataract surgery, as well as its influence on the onset of anaesthesia and akinesia. The safety of sodium bicarbonate as an adjunct to lignocaine has been demonstrated in a study by Gupta and Kapoor [9].

However, further research is needed to clarify the differences between sodium bicarbonate and hyaluronidase as adjuncts in peribulbar anaesthesia. Given the limited literature, this study was designed to provide a comparative evaluation of the two agents and to explore the potential of sodium bicarbonate as a readily available, cost-effective alternative for ocular anaesthesia.

The primary objective of this study was to compare the efficacy of sodium bicarbonate and hyaluronidase as adjuvants to lignocaine-bupivacaine in terms of pain during the administration of peribulbar block for cataract surgery. The secondary objectives were to assess the onset of ocular anaesthesia and the onset of ocular akinesia.

## Materials and methods

This interventional, randomised, comparative clinical study was conducted over 18 months, from May 2023 to November 2024. The study was initiated following approval from the Institutional Ethical Committee (Ref. No: SU/SMS&R/76-A/2023/140, dated 04/05/2023). All procedures involving human participants adhered to the ethical standards of the Institutional Review Board and the principles of the Declaration of Helsinki (1975, revised 2000). Written informed consent was obtained from all participants after explaining the nature, purpose, potential risks, and benefits of the study in their native language. Patient confidentiality was strictly maintained, and participation was entirely voluntary, with the option to withdraw at any stage without affecting medical care.

Patients aged 40-70 years scheduled for cataract surgery under peribulbar anaesthesia were eligible for inclusion. Exclusion criteria were a history of ocular surgery in the same eye, ocular trauma, or known allergy to lignocaine or hyaluronidase. Following consent, each patient underwent a detailed evaluation, including medical, ocular, and allergy history, along with a comprehensive ophthalmic examination.

**Figure 1:**
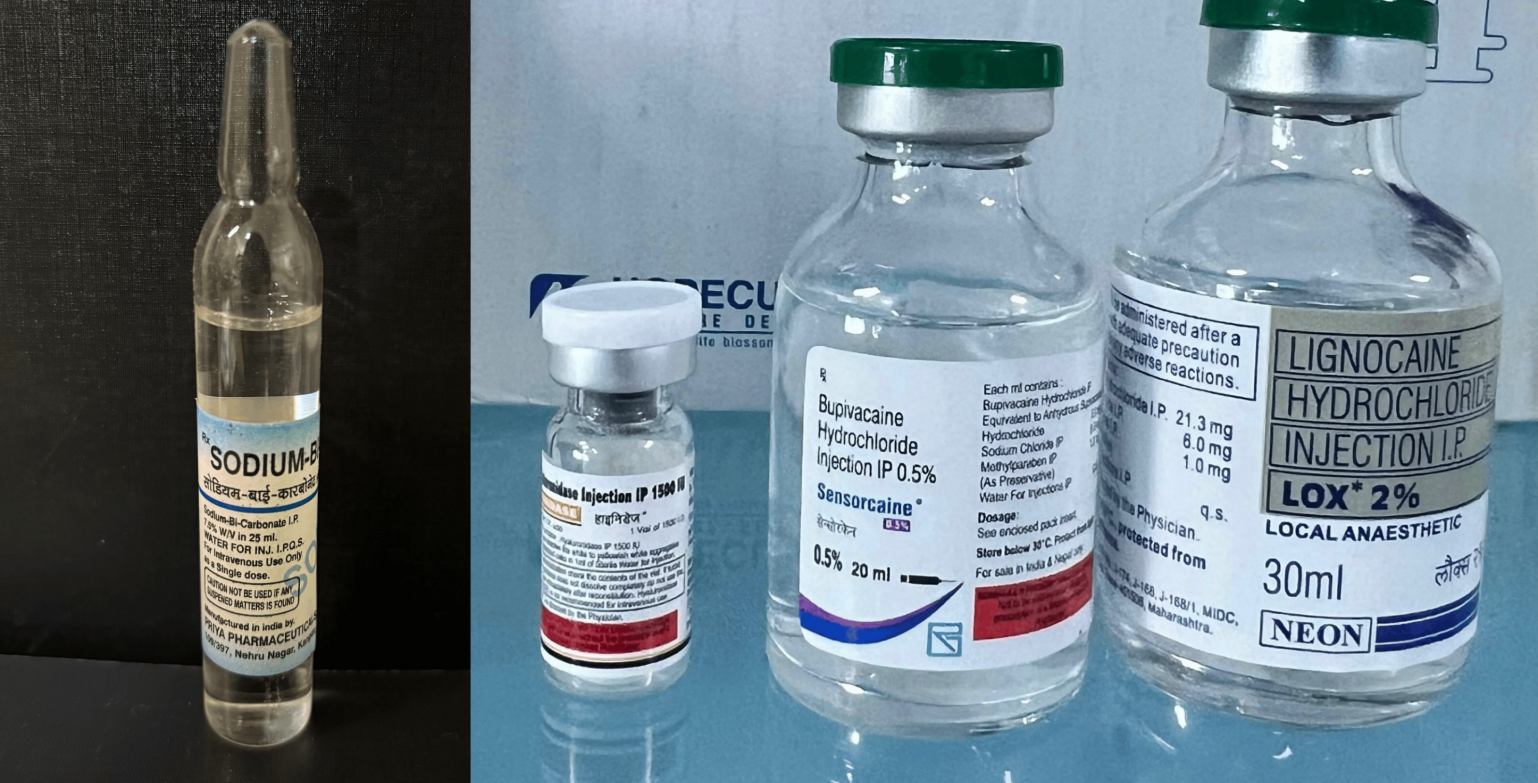
Vials of sodium bicarbonate, hyaluronidase, bupivacaine, and lignocaine

Participants were randomly allocated into two groups using a computer-generated randomisation table: Group A (hyaluronidase group), 2% lignocaine (5 mL) + 0.5% bupivacaine (5 mL) + hyaluronidase (1500 IU in 30 mL lignocaine); and Group B (sodium bicarbonate group), 2% lignocaine (5 mL) + 0.5% bupivacaine (5 mL) + 7.5% sodium bicarbonate (1 mL in 30 mL lignocaine) [9] (Figure 1).

**Figure 2:**
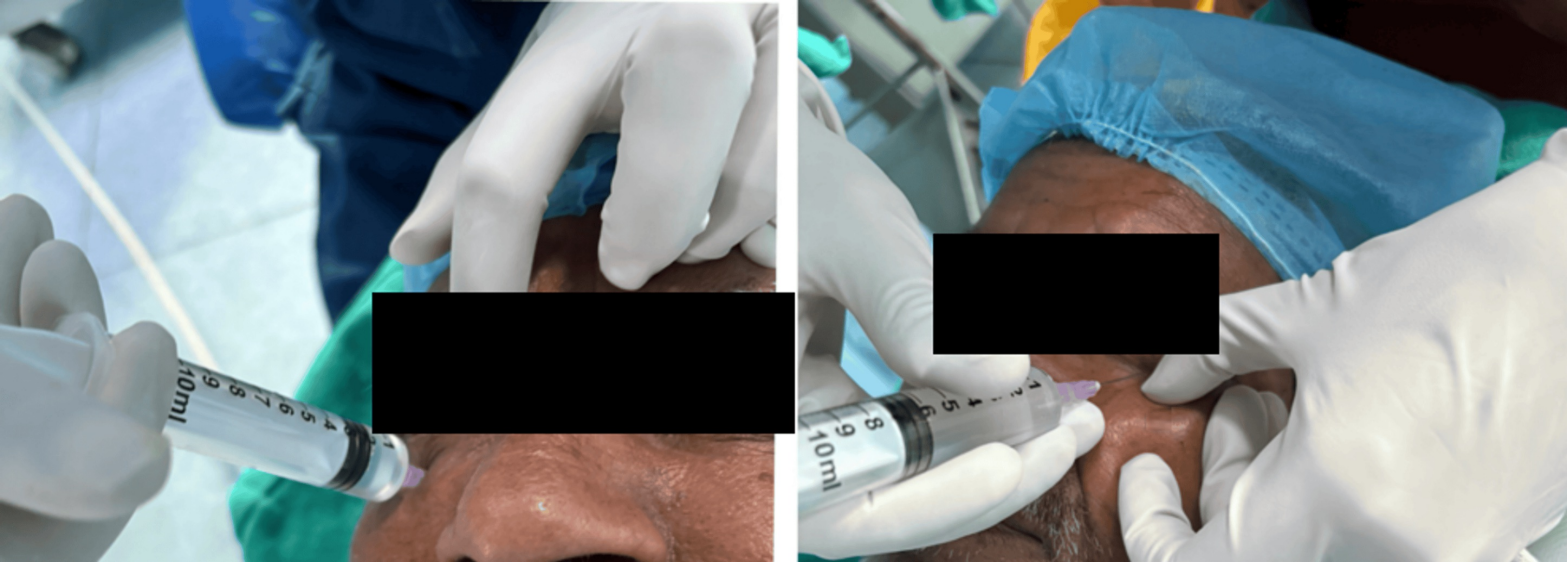
Injection process of peribulbar anaesthesia

All injections were administered by the same experienced operator using a single-point peribulbar technique with a 24-G needle. A total of 6 mL of the assigned solution was injected at the junction of the medial two-thirds and lateral one-third of the lower eyelid, with the eye in the primary gaze position (Figure 2). The injection time was recorded using a stopwatch. 

The anaesthetic solutions were prepared by an investigator not involved in outcome assessment. Both patients and the assessor were blinded to group allocation. Pain (Visual Analogue Scale (VAS)), degree of anaesthesia, and akinesia were assessed by an independent observer who was blinded to the intervention.

Pain intensity was evaluated using the VAS [10,11]. The onset of anaesthesia was determined by testing corneal and conjunctival sensation with a cotton wick, while the onset of akinesia was recorded and graded every minute for 10 minutes.

Akinesia and anaesthesia were classified into three grades: Grade 1, complete anaesthesia and akinesia, with total absence of eye movements, full corneal and conjunctival anaesthesia, and painless insertion of the superior rectus bridle suture (where applicable); Grade 2, adequate anaesthesia and akinesia for safe intraocular surgery, with eye movements <15° in any direction, complete corneal and conjunctival anaesthesia, and painless insertion of the superior rectus bridle suture if required; Grade 3, unsuccessful anaesthesia and akinesia, characterised by eye movements >15° in any gaze, despite complete corneal and conjunctival anaesthesia.

Data were compiled in Microsoft Excel (Microsoft Corp., Redmond, WA, USA) and analysed using IBM SPSS Statistics for Windows, Version 22.0 (Released 2013; IBM Corp., Armonk, NY, USA). Normality of quantitative variables was assessed using the Kolmogorov-Smirnov test. Quantitative data were expressed as mean ± standard deviation, and qualitative data as numbers and percentages with graphical representation. Associations between qualitative variables were analysed using Pearson’s chi-square test, while group comparisons for quantitative variables were performed with the independent t-test. A p-value ≤ 0.05 was considered statistically significant.

## Results

The study included 80 participants, with 40 each in Group A (lignocaine with hyaluronidase) and Group B (lignocaine with sodium bicarbonate). The sample size was calculated a priori based on the difference in mean onset of akinesia reported by Sodani et al. [10]. At a 95% confidence level (two-sided α = 0.05) with 80% power, a minimum of 35 participants per group was required. To account for attrition, 40 participants were enrolled in each group (total n = 80), providing an achieved power of approximately 97%-98%.

The mean age of participants was 59.7 ± 6.2 years in Group A and 59.0 ± 7.5 years in Group B. The difference in age distribution was not statistically significant (p = 0.662), indicating comparability between groups. Gender distribution was also similar: Group A included 21 (52.5%) females and 19 (47.5%) males, while Group B had 19 (47.5%) females and 21 (52.5%) males. This difference was statistically non-significant (p = 0.655), minimising the risk of gender-related confounding.

Baseline vital signs prior to block administration were comparable between groups. Group A demonstrated a mean pulse rate of 74.8 ± 7.5 beats per minute, SBP 125.8 ± 12.6 mmHg, DBP 77.3 ± 8.7 mmHg, and RR 14.5 ± 0.9 breaths per minute. Group B showed values of 72.2 ± 8.9 beats/min, SBP 124.6 ± 15.6 mmHg, DBP 75.3 ± 10.1 mmHg, and RR 14.5 ± 0.8 breaths/min. None of these differences were statistically significant (p > 0.05), confirming hemodynamic stability and baseline comparability.

Pain perception, assessed using VAS, demonstrated a statistically significant difference between the groups. In Group A, 25 (62.5%) participants reported scores of 6-7 (moderate-to-severe pain), while 15 (37.5%) reported scores of 4-5; no participant reported a score <4. In contrast, Group B had lower pain scores, with 29 (72.5%) participants scoring 4-5, 7 (17.5%) scoring 2-3, and only 4 (10.0%) reporting scores of 6-7. Chi-square analysis revealed a significant difference in VAS distribution between the groups (χ² = 26.61, p = 0.01), indicating reduced pain perception with the sodium bicarbonate mixture (Table 1).

**Table 1:** Comparison of Visual Analogue Scale (VAS) scores between groups

		Group A		Group B		Chi-square (p-value)
		Frequency (n)	Percent	Frequency (n)	Percent	
VAS	0-1	0	0.0%	0	0.0%	26.61 (0.01)*
	2-3	0	0.0%	7	17.5%	
	4-5	15	37.5%	29	72.5%	
	6-7	25	62.5%	4	10.0%	
	8-10	0	0.0%	0	0.0%	

The onset of anaesthesia was significantly faster in Group B (1.99 ± 0.63 minutes) compared to Group A (2.32 ± 0.70 minutes; p = 0.02). In contrast, the onset of akinesia was faster in Group A (5.22 ± 1.21 minutes) than in Group B (6.35 ± 0.90 minutes; p = 0.01). These findings, presented in Table 2, indicate that sodium bicarbonate accelerates sensory block, whereas hyaluronidase facilitates earlier motor blockade.

**Table 2:** Comparison of mean onset of anaesthesia and onset of akinesia between the groups

	Group A		Group B		p-value
	Mean ± SD	Median (IQR)	Mean ± SD	Median (IQR)	
Onset of anaesthesia (mins)	2.32 ± 0.7	2.35 (1.16-3.47)	1.99 ± 0.63	2.24 (1.11-3.12)	0.02*
Onset of akinesia (mins)	5.22 ± 1.21	5.21 (3.39-7.78)	6.35 ± 0.90	6.28 (4.71-7.82)	0.01*

All patients in both groups achieved Grade 1 anaesthesia, indicating complete sensory blockade. With respect to akinesia, 33 (84.6%) participants in Group A attained Grade 1 (complete akinesia), compared to 28 (70.0%) in Group B. Conversely, Grade 2 akinesia was observed in 12 (30.0%) patients in Group B versus 6 (15.4%) in Group A (Table 3). The difference in akinesia grades between the groups was not statistically significant (p = 0.12).

**Table 3:** Comparison of the grade of anaesthesia and akinesia between the groups

		Group A		Group B		Chi-square (p-value)
		Frequency (n)	Percent	Frequency (n)	Percent	
Grade of anaesthesia	1.0	40	100.0%	40	100.0%	-
Grade of akinesia	1.0	33	84.6%	28	70.0%	2.39 (0.12)
	2.0	6	15.4%	12	30.0%	

Minimal complications were observed in both groups. Chemosis occurred in 6 (15.0%) patients in Group A and 5 (12.5%) patients in Group B, with no statistically significant difference (p = 0.74), as shown in Table 4. No other significant complications were reported, reaffirming the overall safety of both adjuncts when combined with lignocaine in peribulbar anaesthesia.

**Table 4:** Comparison of the presence of complications between the groups

		Group A		Group B		Chi-square (p-value)
		Frequency (n)	Percent	Frequency (n)	Percent	
Complications, if any	Chemosis	6	15.0%	5	12.5%	0.105 (0.74)
	None	34	85.0%	35	87.5%	

## Discussion

Cataract surgery is among the most frequently performed ophthalmic procedures, and effective regional anaesthesia is essential for patient comfort and optimal surgical outcomes. The peribulbar block, which involves injecting local anaesthetic around the globe to achieve both anaesthesia and akinesia, is widely preferred for its favourable safety profile compared to the retrobulbar block. Lignocaine is commonly used for peribulbar anaesthesia but often requires adjuvants to enhance its efficacy, shorten onset time, and prolong duration.

Hyaluronidase has long been employed as an adjunct because it enhances drug diffusion by breaking down components of the extracellular matrix, resulting in faster onset and improved spread of anaesthesia. More recently, attention has shifted to alkalinisation with sodium bicarbonate, which increases the pH of lignocaine. This converts a greater proportion of the drug into its non-ionised form, allowing quicker tissue penetration and reducing pain on injection.

The present study aimed to compare the effectiveness of sodium bicarbonate and hyaluronidase as adjuncts to lignocaine in peribulbar block during cataract surgery. Key outcomes evaluated included pain perception (VAS score), onset of anaesthesia, onset of akinesia, and safety profile, to determine which adjuvant offers superior clinical benefit. These findings add to the ongoing discussion on optimising regional anaesthesia techniques for cataract surgery, ensuring both patient comfort and surgical efficacy.

A total of 80 participants were included, with 40 in each group. Group A received a local anaesthetic mixture with hyaluronidase, while Group B received lignocaine with sodium bicarbonate. The results were analysed and compared with existing literature to assess the effectiveness and safety of sodium bicarbonate as an alternative to hyaluronidase in peribulbar anaesthesia.

The demographic parameters, including mean age and gender distribution, were comparable between the groups, with no statistically significant differences. This finding is in line with Sodani et al., who also reported a similar age distribution and no significant differences in baseline demographics between groups [10]. Likewise, Unnisa and Racha found no significant variation in demographic factors between patients receiving sodium bicarbonate and hyaluronidase as adjuncts to peribulbar anaesthesia [12].

Vital parameters such as pulse rate, blood pressure, and respiratory rate were also similar between the two groups, with no significant differences. These results are consistent with Gupta and Kapoor, who reported that neither sodium bicarbonate nor hyaluronidase significantly affected systemic hemodynamic parameters, further supporting their safety in peribulbar anaesthesia [9].

A key finding of the present study was that the mean VAS score was lower in Group B, indicating reduced pain perception with sodium bicarbonate compared to hyaluronidase. This observation is consistent with Sodani et al., who reported significantly lower mean pain scores in patients receiving sodium bicarbonate (5.12 ± 1.17) compared to those receiving hyaluronidase (7.16 ± 1.09) [10].

The onset of anaesthesia was also significantly faster in Group B (p < 0.05), which agrees with the findings of Srinivasan et al. [13] and Gupta and Kapoor [9], both of whom demonstrated that buffering lignocaine with sodium bicarbonate shortened the onset time of anaesthesia. Similarly, Unnisa and Racha reported that sodium bicarbonate achieved a quicker onset of block compared to hyaluronidase [12].

Conversely, the mean onset time of akinesia was significantly longer in Group B compared to Group A (p < 0.05), indicating that hyaluronidase was more effective in achieving early and complete akinesia. This aligns with the findings of Nazareth et al., who observed that while sodium bicarbonate accelerated the onset of anaesthesia, the akinetic effect was more pronounced in patients receiving hyaluronidase [14]. The superior effect of hyaluronidase can be explained by its “spreading effect,” which involves depolymerising hyaluronic acid in the extracellular matrix and thereby facilitating rapid, uniform diffusion of the anaesthetic to the motor nerves of the extraocular muscles [4]. In contrast, sodium bicarbonate enhances the proportion of non-ionised anaesthetic molecules through alkalinisation, promoting faster nerve penetration and sensory block but without improving tissue permeability. This likely accounts for the delayed and less consistent motor block observed in Group B [7]. Clinically, a slower onset of akinesia may necessitate a longer waiting period before surgery can begin, potentially prolonging operating theatre time and reducing workflow efficiency. In high-volume cataract surgery settings, this delay could impact surgeon comfort and patient turnover.

Complications between the two groups were not significantly different, consistent with previous findings. Unnisa and Racha similarly reported that both sodium bicarbonate and hyaluronidase demonstrated comparable safety profiles, with no significant differences in adverse effects [12].

From a cost perspective, sodium bicarbonate represents a cheaper and more readily available alternative to hyaluronidase, which may be particularly advantageous in resource-limited settings. However, this benefit must be balanced against its relatively delayed onset of akinesia, which could limit its usefulness in high-volume surgical centres where efficiency is crucial.

This study has several limitations that warrant consideration. The relatively small sample size of 80 participants may reduce the statistical power. Being a single-centre study, the findings may not be generalisable across different populations or healthcare systems. In addition, the short follow-up period restricted the assessment of outcomes to the immediate perioperative phase, excluding potential delayed complications or long-term patient satisfaction. Another limitation was the lack of evaluation of block duration. While onset times provide useful information on the rapidity of block effectiveness, the duration of anaesthesia and akinesia is also clinically important for both intraoperative comfort and postoperative analgesia. Future multicentre studies with larger cohorts, longer follow-up, and comprehensive outcome measures, including both onset and duration, are recommended to validate and build upon these findings.

## Conclusions

Overall, the present study confirms that sodium bicarbonate is an effective alternative to hyaluronidase in peribulbar anaesthesia, particularly by reducing pain perception and hastening the onset of anaesthesia. However, hyaluronidase remains superior in achieving faster akinesia. Considering its affordability and accessibility, sodium bicarbonate can be regarded as a practical adjunct for peribulbar blocks in cataract surgery. In resource-limited settings such as India, where cost plays a crucial role in surgical planning, sodium bicarbonate provides an economical option to enhance block efficacy without significantly increasing procedural expenses.
